# Pioglitazone alleviates cisplatin nephrotoxicity by suppressing mitochondria‐mediated apoptosis via SIRT1/p53 signalling

**DOI:** 10.1111/jcmm.15782

**Published:** 2020-09-02

**Authors:** Jiong Zhang, Yang Zou, Yan Cheng‐Jing, Lu Xiang‐Heng, Xue‐Peng Wang, Xiao‐Jia Yu, Gui‐sen Li, Jia Wang

**Affiliations:** ^1^ Department of Nephrology Sichuan Clinical Research Center for Kidney Disease Sichuan Academy of Sciences & Sichuan Provincial People's Hospital University of Electronic Science and Technology Chengdu China; ^2^ Department of Nephrology The First People's Hospital of Liangshan Yi Autonomous Prefecture Xichang China; ^3^ Queen Mary Colleges Medical College of Nanchang University Nanchang China; ^4^ Sichuan Academy of Sciences & Sichuan Provincial People's Hospital General Medicine Center and University of Electronic Science and Technology Chengdu China

**Keywords:** cisplatin nephrotoxicity, mitochondrial, Pioglitazone, SIRT1

## Abstract

Pioglitazone (PIO) attenuates cisplatin nephrotoxicity whereas the underlying mechanism remains unknown. Apoptosis is associated with mitochondrial dysfunction and SIRT1 activation can decrease cell apoptosis in cisplatin nephrotoxicity. Therefore, we explored whether the protective effect of PIO in cisplatin nephrotoxicity is achieved by suppressing mitochondria‐mediated apoptosis through SIRT1/p53 signalling regulation. Cell viability, apoptosis, survival rate, renal pathology and function were examined. Moreover, we also analysed the expression of SIRT1, Acetyl‐p53, mitochondrial membrane potential (MMP), reactive oxygen species (ROS), mitochondrial permeability transition pore (mPTP) opening, adenosine triphosphate (ATP) and apoptosis‐related protein in vivo and in vitro. Pioglitazone treatment significantly increased cell viability, promoted SIRT1‐p53 interaction, upregulated Bcl‐2 expression, activated SIRT1 and elevated mitochondrial ATP synthesis after cisplatin treatment. However, PIO decreased the generation of ROS, opening of mPTP, dissipation of MMP and translocation of cytochrome c after cisplatin treatment. Pioglitazone also reduced the activation of caspase‐3 and caspase‐9, lowered the ratio of Bax/Bcl‐2, attenuated kidney pathological damage and dysfunction, down‐regulated the expression of Acetyl‐p53, PUMA‐α and Bax and abated cell apoptosis after cisplatin treatment. The SIRT1 inhibitor, EX527, clearly reversed the protective effects of PIO. These results implied PIO attenuated cisplatin nephrotoxicity by suppressing mitochondria‐mediated apoptosis through regulating SIRT1/p53 signalling.

## 1 INTRODUCTION

Cisplatin, a classic chemotherapeutic drug, is widely used in clinics to treat various solid organ malignant tumours.^1^ However, as a coin has two sides, cisplatin also often has serious side effects in the treatment of tumours. For example, cisplatin induces acute kidney injury (AKI),^1^, ^2^ an important leading cause of acute renal failure (ARF) and death in humans worldwide.^2^, ^3^ Cisplatin treatment damages the structure and function of kidneys, causing kidney dysfunction, tubular atrophy, epithelial cell apoptosis, necrosis and other physiologic perturbation.^4^ It is widely accepted that p53‐mediated mitochondrial dysfunction plays a vital role in apoptosis of renal tubule cells in cisplatin nephrotoxicity, via a mechanism associated with rapid mitochondrial membrane permeabilization (MMP), increased mitochondrial permeability transition pore (mPTP) opening, higher ATP degradation, caspase‐3 activation and cytochrome c release.^5^, ^6^

Bcl‐2 family proteins are the switches of mitochondrial apoptosis,^7^ which regulate mPTP opening, the key rate‐limiting event during apoptosis induction.^8^ In addition, cysteine aspartic proteases, which belong to the caspase family, play vital roles in initiating and completing cell apoptosis and are markers for cellular apoptosis.^9^ For example, caspase‐3 plays an important role in activating DNA breakage in apoptotic cells.^9^ Some studies have shown that cytochrome c, a pro‐apoptotic signal, initiates a caspase amplification cascade, leading to the cleavage of a series of proteins.^10^, ^11^ It is noteworthy that anti‐apoptotic members of the Bcl‐2 family protect mitochondrial integrity mainly by restricting cytochrome c release and suppressing cell separation.^10^, ^11^ Therefore, promoting anti‐apoptosis would be an effective strategy against cisplatin nephrotoxicity.

An important member of sirtuins, SIRT1 deacetylates various substrates including p53.^12^, ^13^ In cisplatin nephrotoxicity, acetylation of p53 can change mitochondrial outer membrane permeabilization in pro‐apoptotic members of the Bcl‐2 family, resulting in the release of cytochrome c and caspase activation.^6^, ^14^

Pioglitazone (PIO), a thiazolidinedione used as an antidiabetic drug, is a SIRT1 agonist that can significantly abate cisplatin nephrotoxicity,^15^ although the underlying mechanism is not yet clear. Therefore, we sought to investigate whether (a) the protective effect of PIO in cell culture and in mouse kidneys was associated with decreasing cell apoptosis due to SIRT1 activation after cisplatin treatment; (b) anti‐apoptosis due to SIRT1 activation was caused by the suppression of p53‐mediated mitochondrial abnormality resulting in cisplatin nephrotoxicity; and (c) p53 deacetylation was involved in SIRT1 activation and p53 inactivation, by promoting SIRT1 and p53 interaction in cisplatin nephrotoxicity.

## 2 MATERIALS AND METHODS

### 2.1 Reagents

Pioglitazone (PIO, purity > 99%), cisplatin (Cis, purity > 99%) and Sirtinol (EX527) were purchased from Sigma (USA) and Selleckchem (USA), respectively. Anti‐SIRT1, anti‐p53, anti‐acetyl‐p53, anti‐cytochrome c, anti‐PUMA‐α, anti‐Bcl‐2, anti‐Bax, anti‐Cleaved‐caspase‐3, anti‐Cleaved‐caspase‐9, anti‐β‐actin and IgG secondary antibodies were all purchased from Cell Signaling Technology (USA). SIRT1 and Caspase‐3 activity kits were purchased from eBioscience. Human tubular epithelial cells (HK‐2) were purchased from ATCC.

### 2.2 Animal experiments

40 Male SPF C57BL/6 mice (8 weeks old, weighing 22‐25 g) were purchased from the West China Experimental Animal Center of Sichuan University and housed in a standard laboratory animal facility with ad libitum water and food. The animal experiment protocol was authorized and approved by the Ethics Committee of Sichuan Provincial People's Hospital and conducted in line with the national ethical standards for experimental animals. 40 C57BL/6 mice were randomly equally divided into Control (Con), Cisplatin (Cis), Pioglitazone (PIO) and EX527(EX) groups. Every group has 10 mice. The cisplatin dose (20 mg/kg) and intervention time (72 hours) were based on previous research.^15^ Pioglitazone was administered by gavage once per day for seven consecutive days at a dose of 40 mg/kg, followed by intraperitoneal injection of cisplatin.^15^ Sirtinol (EX527, 10 mg/kg) and resveratrol were administered simultaneously once per day for 7 days by intraperitoneal injection.^14^ At 72 hours after cisplatin injection, mice were euthanized under anaesthesia to collect serum and kidney tissue.

### 2.3 Cell culture

Immortalized human renal tubular epithelial cells (HK‐2) were cultured in low glucose complete DMEM medium supplemented with foetal calf serum (10%) and antibiotics (0.1 mg/mL streptomycin and 100 U/mL penicillin G. HK‐2 were seeded in a six‐well tissue culture plate and incubated for 16 hours at 37°C with 5% CO_2_ to allow adherence and subsequently serum starved. To determine the effect of inhibiting SIRT1, cells were pre‐treated with EX527 for 30 minutes and then incubated with/without PIO (40 µg/mL) for 30 minutes. The dose of EX527 was based on the previous work.^14^ Thereafter, HK‐2 cells were treated with cisplatin for 8 hours.

### 2.4 Biochemical parameters

To evaluate renal function, sera collected from all groups were centrifuged and the concentrations of creatinine and urea nitrogen (BUN) were determined in the core laboratory of Sichuan Provincial People's Hospital using standard assay kits (Google biology).

### 2.5 Histological examination

Harvested renal tissue was fixed in 10% formalin and then dehydrated, cleared, waxed, embedded, sectioned, dewaxed and haematoxylin‐eosin (HE)–stained. The sections were observed at 400× magnification on a light microscope. The microscopic total damage score was determined according to the degree of tubular necrosis, dilatation, haemorrhage, cast formation and cell lysis as follows: 0 for all normal tubules; 1 for 75% necrotic tubules; 2 for 10‐25% necrotic tubules; 3 for 26‐75% necrotic tubules; and 4 for >75% necrotic tubules.^15^

### 2.6 Extraction of mitochondrial protein

Cytosolic and mitochondrial fractions were extracted using the Mitochondria Isolation Kit (Pierce). The proteins were incubated with anti‐cytochrome *c* antibody.

### 2.7 Immunoblot assay

Immunoblot analysis was performed as previously described.^13^ Renal tissues from all groups were homogenized on ice in PBS cocktail (Google biology). HK‐2 cell or kidney samples were incubated overnight at 4°C with rabbit anti‐SIRT1 (1:500), p53 (1:1000), Acetyl‐p53 (1:500), PUMA‐α (1:200), Cleaved‐caspase‐3 (1:500), Cleaved‐caspase‐9 (1:400), Bcl‐2 (1:1000), Bax (1:1000), cytochrome c (1:400), β‐actin (1:4000) and sequentially, with secondary antibody (1:3000). Next, the membrane was rinsed and enhanced with chemiluminescent reagent (Zhongshan Gold Bridge). All band signals were analysed with densitometry scanning (LAS).

### 2.8 Mitochondrial ATP synthesis assay

Adenosine triphosphate production was assessed as previously described.^6^ Adenosine triphosphate synthesis was monitored at 340 nm using the Coomassie brilliant blue method according to the Jiancheng ATP enzyme kit protocol.

### 2.9 Measurement of mitochondrial membrane potential (MMP)

To detect MMP, 2 mL of detecting medium was prepared. A 100 μL mitochondria suspension was incubated for 5 minutes at 25°C, followed by the addition of CaCl_2_ (100 nmoL final concentration). After the mitochondria were swollen, the supernatant was extracted by centrifugation for 5 minutes, and then, the fluorescence value was measured. The emission wavelength was set at 525 nm and excitation wavelength at 500 nm. MMP levels were deduced from the ratio of green and red fluorescence intensity.

### 2.10 Reactive oxygen species (ROS) measurement

Kidney and HK‐2 cell homogenates were mixed with dichlorofluorescin diacetate (DCFH) and incubated at 37°C in water bath for 10 minutes. Fluorescence intensity was measured using a DCFH fluorescence spectrophotometer, and the expression of reactive oxygen species (ROS) in kidneys was inferred from the relative fluorescence intensity (RFI).

### 2.11 Assessment of mPTP opening

The opening of mPTP was determined by monitoring the change in mitochondrial optical density assessed by absorbance measurements at 520 nm wavelength as previously described.

### 2.12 SIRT1 activity assay

SIRT1 activity was measured using the SIRT1 Activity Fluorescent Assay Kit (Beyotime, China) according to the manufacturer's instructions. The emission and excitation wavelengths were set at 460 nm and 360 nm.

### 2.13 Caspase‐3 activity assay

Caspase‐3 activity was measured using the caspase‐3 Activity Fluorescent Assay Kit (Beyotime, China) according to the manufacturer's instructions. The emission wavelength was set at 405 nm.

### 2.14 Apoptosis and cell viability assays

Apoptosis in kidneys was measured using the terminal deoxynucleotidyl transferase‐mediated uridine triphosphate (dUTP) nick‐end labelling (TUNEL) assay kit (Google biology, Wuhan) according to the manufacturer's instructions. The number of apoptotic cells in the kidney in each group was evaluated by counting TUNEL‐positive renal cells in each microscope field of view at 400× magnification. The viability of HK‐2 cells was measured using the CCK‐8 assay kit (Google biology, Wuhan), according to the manufacturer's instructions.

### 2.15 Statistical analysis

All data were expressed as mean ± SEM. Multiple comparisons were performed using the t test or one‐way ANOVA. Individual means were compared using Tukey's post hoc test. *P* values < .05 were considered statistically significant.

## 3 RESULTS

### 3.1 Cisplatin reduces SIRT1 activation in HK‐2 cell

**FIGURE 1:**
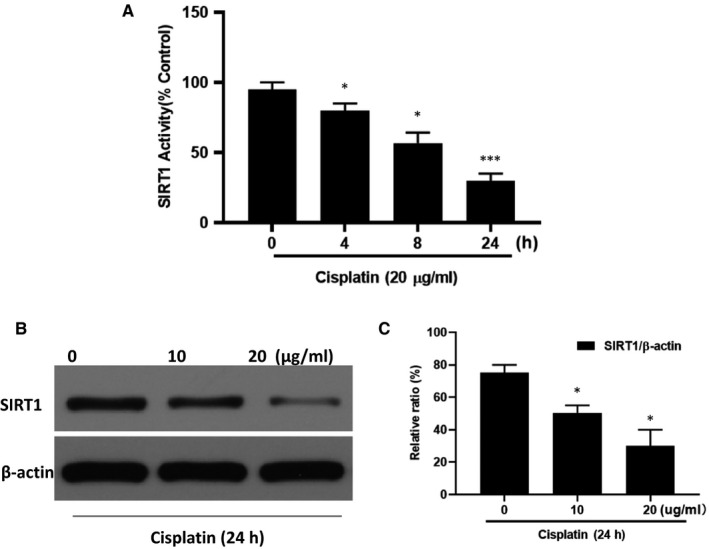
The effect of cisplatin on the activation of SIRT1 in HK‐2 cells. A, The effect of cisplatin on SIRT1 activity was examined using the SIRT1 Activity Kit. B, A representative SIRT1 Western blot analysis strip. C, Semi‐quantitative analysis of the relative amounts of SIRT1 in each group of mice. Bars represent the mean ± SEM (n = 10) ^*^ *P* < .05, ^**^ *P* < .01 (Cis vs Con). Cis, cisplatin; Con, control

To test the effects of cisplatin on SIRT1 activation, we evaluated the expression and activity of SIRT1 in HK‐2 cells. The cells were incubated with cisplatin (20 µg/mL) for 4, 8 and 24 hours (Figure 1A) with the maximum invention time set at 24 hours. Cisplatin treatment markedly reduced SIRT1 activity. Moreover, cisplatin decreased SIRT1 expression in a dose‐dependent manner. At the maximum cisplatin dose (20 µg/mL), SIRT1 expression was decreased by approximately 40% (Figure 1B).

### 3.2 PIO decreases cisplatin‐induced cytotoxicity and apoptosis of HK‐2 cells, and via SIRT1

**FIGURE 2:**
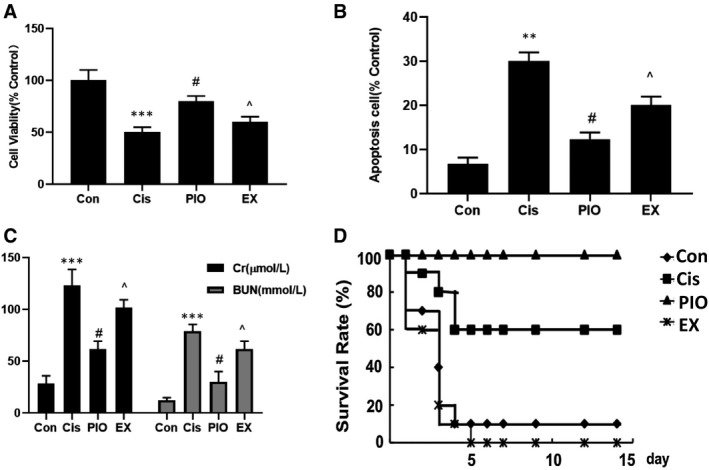
The effect of PIO on cell viability and apoptosis, and mice renal function and survival rate in cisplatin nephrotoxicity. A, The effect of PIO on cisplatin‐induced cell viability was examined using CCK‐8. B, Flow cytometry analysis of the effect of PIO on cisplatin‐induced cell apoptosis. C, The effect of PIO on the levels of serum Cr and BUN in cisplatin nephrotoxicity; D, The effect of PIO on mice survival rates in cisplatin nephrotoxicity. Bars represent the mean ± SEM (n = 10). ^***^ *P* < .001, (Cis vs Con); ^#^ *P* < .05 (PIO vs Cis); ^^^ *P* < .05 (EX vs PIO). Cis, cisplatin; Con, control; PIO, Pioglitazone; EX, EX527

To evaluate the role of SIRT1 in cisplatin‐induced cell cytotoxicity and apoptosis, HK‐2 cells were treated with cisplatin and PIO in the absence or presence of EX527 and analysed with CCK‐8 and flow cytometry. This analysis showed that cisplatin decreased cell viability and increased apoptosis of HK‐2 cells. Pre‐treatment with PIO significantly increased cell viability and reduced the apoptosis of cisplatin‐treated HK‐2 cells (Figure 2A,B). Compared to PIO, EX527 more remarkably decreased cell cytotoxicity and increased cell apoptosis after cisplatin treatment (Figure 2A,B).

### 3.3 PIO ameliorates renal dysfunction and improves survival rate in cisplatin‐induced nephrotoxicity though SIRT1

To further evaluate the role of SIRT1 in renal function, we examined the effect of PIO, a SIRT1 agonist, on the expression of the classic renal function markers, creatinine (Cr) and BUN,^15^ after cisplatin‐induced nephrotoxicity. Mice treated with cisplatin had higher expression of serum Cr and BUN than mice treated with the vehicle (Figure 2C). Pre‐treatment with PIO significantly attenuated cisplatin‐induced increase of serum Cr and BUN. However, the protective effect of PIO on serum Cr and BUN was offset by EX527. To further demonstrate the protective role of SIRT1 activation in cisplatin nephrotoxicity, we examined the effect of PIO on mice survival rates. In a previous study, the dose of cisplatin that induced acute kidney failure was found to be 30 mg/kg.^15^ At this dose, the survival rate of cisplatin‐only group was approximately 20%, which was lower than that of the PIO group, which was approximately 80%. These results indicated that PIO could improve survival rates in cisplatin‐induced ARF. However, EX527 reversed the protective effects of PIO, decreasing the survival rate to approximately 10% (Figure 2C,D).

### 3.4 PIO decreases renal histological injury and apoptosis after cisplatin nephrotoxicity via SIRT1

**FIGURE 3:**
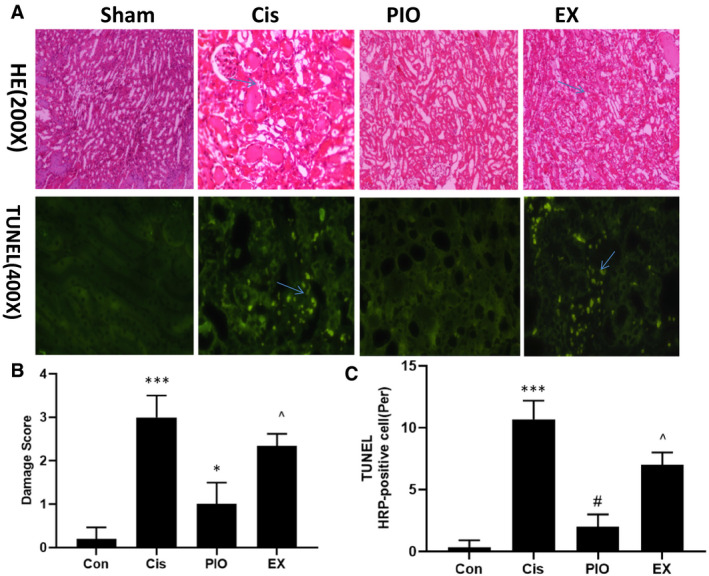
The effect of PIO on pathological kidney morphology and cell apoptosis in cisplatin nephrotoxicity. A, HE staining and TUNEL were used to observe pathological kidney morphology (200× magnification) and cell apoptosis (400× magnification) in cisplatin nephrotoxicity. B, Semi‐quantitative assessment of the pathological injury morphology in the kidneys. C, Semi‐quantitative assessment of cell apoptosis. Bars represent the mean ± SEM (n = 10). ^***^ *P* < .001, (Cis vs Con); ^#^ *P* < .05 (PIO vs Cis); ^^^ *P* < .05 (EX vs PIO). Cis, cisplatin; Con, control; PIO, Pioglitazone; EX, EX527

To further demonstrate the protective effect of SIRT1 activation on cisplatin treatment, we measured renal pathological morphology and cell apoptosis using HE staining and TUNEL, respectively. HE staining showed greater renal pathological injury in mice treated with cisplatin, characterized with tubular epithelial cell swelling, desquamation and vacuolization, tubular necrosis dilation, and brush border loss with damage score (3 ± 0.5) points in Cis group and (0.2 ± 0.1) points in Con group as shown in Figure 3A,B. Pre‐treatment with PIO significantly alleviated cisplatin‐induced increase in renal pathological injury, and lowered tubular epithelial cell swelling, desquamation and vacuolization, tubular necrosis dilation and brush border loss with damage score (1 ± 0.5) points in PIO group. Compared with PIO group, the EX group had more serious cisplatin‐induced renal pathological injury with damage score (2 ± 0.5) points in EX group (Figure 3A,B). TUNEL indicated that mice treated with cisplatin had more TUNEL‐positive renal cells (Figure 3A‐C). Treatment with PIO significantly reduced cisplatin‐induced increase in TUNEL‐positive renal cells. However, the EX group had more TUNEL‐positive renal cells than the PIO group, indicating that the suppressive effect of PIO on cell apoptosis could be significantly attenuated by EX527 (Figure 3A‐C).

### 3.5 PIO inhibits cisplatin‐induced acetylation of p53 by promoting SIRT1‐p53 interaction

**FIGURE 4:**
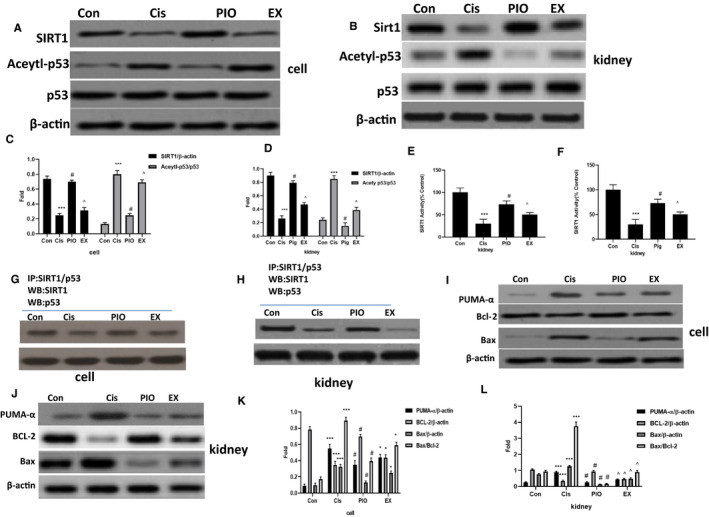
The effect of PIO on SIRT1 activation, p53‐SIRT1 interaction and p53, PUMA‐α, Bcl‐2 and Bax in HK‐2 cells and mice kidneys after cisplatin treatment. A and B, Representative Western blot analysis strips for SIRT1, p53 and Acetylp53 in HK‐2 cells and mice kidneys after cisplatin treatment. C and D, Semi‐quantitative analysis of the relative amounts of SIRT1, p53 and Acetylp53 in HK‐2 cells and mice kidneys. E and F, The effect of PIO on SIRT1 activity in HK‐2 cells and mice kidneys after cisplatin treatment. G and H, Representative co‐immunoprecipitation analysis of p53‐SIRT1 interactions in HK‐2 cells and mice kidneys after cisplatin treatment. I and J, Representative Western blot analysis strips for PUMA‐α, Bcl‐2 and Bax in HK‐2 cells and mice kidneys after cisplatin treatment. K and L, Semi‐quantitative analysis of the relative amounts of PUMA‐α, Bcl‐2 and Bax in HK‐2 cells and mice kidneys. Bars represent the mean ± SEM (n = 10). ^***^ *P* < .001, (Cis vs Con); ^#^ *P* < .05 (PIO vs Cis); ^^^ *P* < .05 (EX vs PIO). Cis, cisplatin; Con, control; PIO, Pioglitazone; EX, EX527

Previous studies have demonstrated that SIRT1/p53 signalling plays a vital role in apoptosis in cisplatin nephrotoxicity.^14^ We tested the effect of PIO on SIRT1 activation, p53 acetylation and p53‐SIRT1 interaction in vivo and in vitro. As shown in Figure 4A‐H, cisplatin significantly decreased SIRT1 expression and activity and SIRT1‐p53 interaction and increased the expression of Acetyl‐p53. However, PIO treatment sharply increased SIRT1 expression and activity, and SIRT1‐p53 interaction, and significantly decreased Acetyl‐p53 expression compared with cisplatin‐only group. EX527 significantly reversed the effect of PIO on cisplatin treatment, as indicated by a lower expression and activity of SIRT1, higher expression of acetylated p53 and lower SIRT1‐p53 interaction in the EX group than in the PIO group.

### 3.6 SIRT1 activation decreases the expression of PUMA‐α and Bax and increases the expression of Bcl‐2 in vivo and in vitro after cisplatin treatment

PUMA‐α, a BH3‐only protein, is a downstream target of p53^16^ and the direct activator of Bax. PUMA‐α can cause mitochondrial dysfunction and release of the apoptotic protein, cytochrome c, by altering Bax/Bcl‐2 ratio, activating executioner caspases and finally leading to cell death.^16^, ^17^ Therefore, we investigated the effect of PIO on the expression of PUMA‐α, Bcl‐2, Bax and on the ratio of Bax/Bcl‐2 in vivo and in vitro after cisplatin intervention. As shown in Figure 4I‐L, cisplatin treatment clearly increased the ratio of Bax/Bcl‐2, upregulated the expression of PUMA‐α and Bax, and down‐regulated the expression of Bcl‐2. Pre‐treatment with PIO significantly decreased PUMA‐α and Bax expression and the Bax/Bcl‐2 ratio, and clearly increased Bcl‐2 expression. EX527 significantly abolished the suppressive effect of PIO on cisplatin treatment, as shown by the higher Bax/Bcl‐2 ratio and PUMA‐α and Bax expression, and lower Bcl‐2 expression in the EX group than in the PIO group.

### 3.7 PIO attenuates cisplatin‐induced mitochondrial abnormalities in vivo and in vitro

**FIGURE 5:**
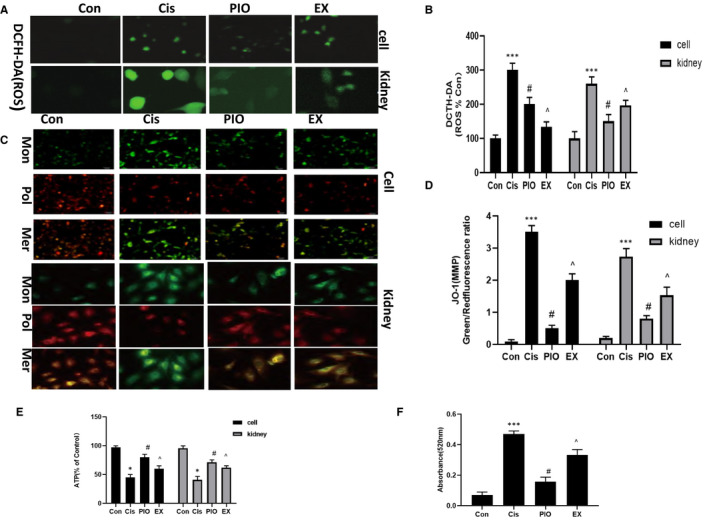
The effect of PIO on mitochondrial abnormalities in HK‐2 cells and mice kidneys after cisplatin treatment. A, Representative DCFH‐DA analysis of ROS production (200× magnification) in HK‐2 cells and mice kidneys after cisplatin treatment. B, A histogram of ROS levels in HK‐2 cells and mice kidneys. DCFH‐DA fluorescence is representative of ROS levels. C, Representative JO‐1 analysis of MMP in HK‐2 cells (200× magnification) and mice kidneys (400× magnification). D, A histogram of MMP levels in HK‐2 cells and mice kidneys. The ratio of green/red fluorescence represents the level of MMP. E, A histogram of mitochondrial ATP synthesis in HK‐2 cells and mice kidneys. F, A histogram of mPTP opening in HK‐2 cells and mice kidneys. The absorbance peak at 520 nm at 20 min represents the level of mPTP opening. Bars represent the mean ± SEM (n = 10). ^***^ *P* < .001, (Cis vs Con); ^#^ *P* < .05 (PIO vs Cis); ^^^ *P* < .05 (EX vs PIO). Cis, cisplatin; Con, control; PIO, Pioglitazone; EX, EX527

Previous study showed that altering the Bax/Bcl‐2 ratio resulted in mitochondrial abnormalities, including the generation of ROS, dissipation of mitochondrial membrane potential (MMP), opening of permeability transition pore (mPTP) and an imbalance of energy in the mitochondrion.^17^ Therefore, we examined ROS levels, MMP, mPTP opening and mitochondrial ATP synthesis in vivo and in vitro. As shown in Figure 5, cisplatin significantly increased the generation of ROS, opening of mPTP and dissipation of MMP and decreased mitochondrial ATP synthesis relative to the controls. However, treatment with PIO significantly reduced the generation of ROS, opening of mPTP and dissipation of MMP, and increased ATP synthesis after cisplatin treatment. SIRT1 inhibition by EX527 significantly abated the suppressive effect of PIO on cisplatin‐induced mitochondrial abnormalities, as shown by lower expression of MMP and ATP, and higher expression of ROS and mPTP opening in the EX group than in the PIO group.

### 3.8 PIO treatment suppresses the release of cytochrome c and the activation of apoptosis cascade reaction after cisplatin treatment

**FIGURE 6:**
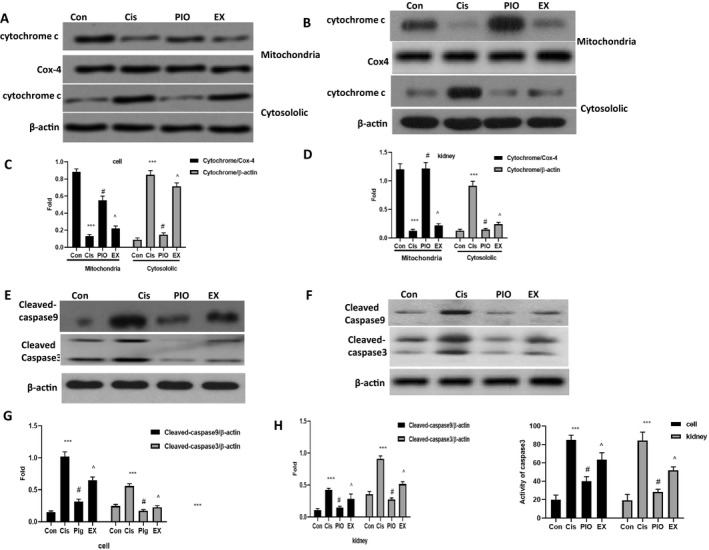
The effect of PIO on the release of cytochrome c, and the activation of caspase‐3 and caspase‐9 in HK‐2 cells and mice kidneys after cisplatin treatment. A and C, Representative Western blot analysis strips for cytochrome c in mitochondrion and the cytoplasm in HK‐2 cells and mice kidneys after cisplatin treatment. B and D, Semi‐quantitative analysis of the relative amounts of cytochrome c in mitochondrion and the cytoplasm in HK‐2 cells and mice kidneys. E and F, Representative Western blot analysis strips for cleaved‐caspase‐3 and cleaved‐caspase‐9 in HK‐2 cells and mice kidneys after cisplatin treatment. G and H, Semi‐quantitative analysis of the relative amounts of cleaved‐caspase‐3 and cleaved‐caspase‐9 in HK‐2 cells and mice kidneys. I, The effect of PIO on caspase‐3 activity in HK‐2 cells and mice kidneys after cisplatin treatment. Bars represent the mean ± SEM (n = 10). ^***^ *P* < .001, (Cis vs Con); ^#^ *P* < .05 (PIO vs Cis); ^^^ *P* < .05 (EX vs PIO). Cis, cisplatin; Con, control; PIO, Pioglitazone; EX, EX527

Mitochondrial abnormalities may lead to the release of cytochrome c from mitochondria into the cytoplasm.^11^, ^16^ To test this assertion, we measured the expression of cytochrome c in mitochondria and cytoplasm in vivo and in vitro after cisplatin treatment and showed that cisplatin treatment led to the translocation of cytochrome c. Compared with controls, cisplatin‐treated cells had higher expression of cytochrome c in the cytoplasm and lower expression in the mitochondria (Figure 6A‐D). Pre‐treatment with PIO significantly suppressed cisplatin‐induced translocation of cytochrome c from mitochondria into cytoplasm. Higher expression of cytochrome c in mitochondria and lower expression in cytoplasm in the PIO group than in the cisplatin‐only group. However, EX527 significantly abolished the suppressive effect of PIO on cisplatin‐induced translocation of cytochrome c, with the EX group showing lower expression of cytochrome c in mitochondria and higher expression in cytoplasm than the PIO group.

Previous studies demonstrated that cytochrome c may cause the activation of caspase‐9 and activate key downstream apoptotic executive protein, caspase‐3, to promote apoptosis.^9^, ^17^ Because caspase‐3 is a key effector protein in the apoptosis protease cascade,^9^, ^17^ we examined the activity of caspase‐3 and the expression of cleaved‐caspase‐3 and cleaved‐caspase‐9 in vivo and in vitro (Figure 6E‐I). The data showed that cisplatin treatment triggered the apoptosis cascade, as indicated by higher caspase‐3 activity and higher cleaved‐caspase‐3 and cleaved‐caspase‐9 expression in the cisplatin group than in the controls. However, PIO treatment sharply decreased caspase‐3 and caspase‐9 activation after cisplatin treatment, as demonstrated by lower caspase‐3 activity and lower cleaved‐caspase‐3 and cleaved‐caspase‐9 expression in the PIO group than in the cisplatin‐only group. EX527 effectively abolished the suppressive effect of PIO on cisplatin‐induced activation of the apoptosis cascade, as confirmed by higher caspase‐3 activity and higher cleaved‐caspase‐3 and cleaved‐caspase‐9 expression in the EX group than in the PIO group.

**FIGURE 7:**
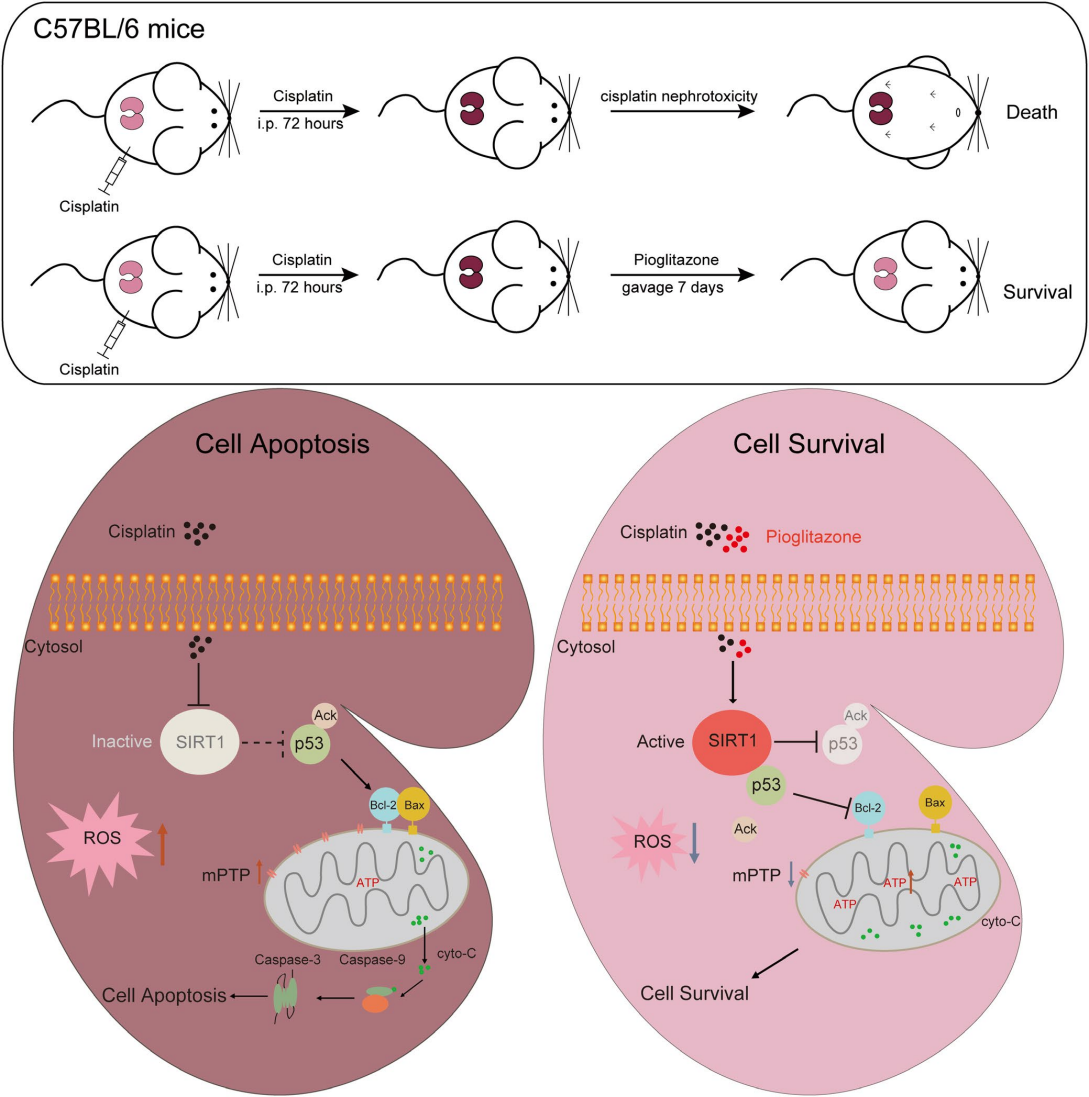
The signal pathway of PIO on mitochondria‐mediated apoptosis in cisplatin nephrotoxicity

The signal pathway of PIO on mitochondria‐mediated apoptosis in cisplatin nephrotoxicity (Figure 7).

## 4 DISCUSSION

SIRT1 is a highly conserved NAD+‐dependent protein deacetylases, which interacts with transcription co‐regulatory factors, chromatin and a variety of important transcription factors.^12^, ^13^ SIRT1 regulates gene transcription through deacetylation and controls many biological processes.^12^, ^13^ In this study, we found that PIO significantly suppressed cisplatin‐induced decrease in the activity and expression of SIRT1 in HK‐2 cells and mouse kidneys. These findings were consistent with the view that SIRT1 plays an important role in cisplatin nephrotoxicity and that PIO is a SIRT1 agonist.^14^, ^15^ We also showed that PIO clearly reduced cell apoptosis and renal pathological injury and renal dysfunction, and increased cell viability and survive rate in vivo and in vitro after cisplatin treatment. Moreover, EX527, a SIRT1 inhibitor, effectively abolished the protective effect of PIO after cisplatin treatment. This observation was supported by data showing higher cell apoptosis and renal pathological damage and renal dysfunction, and less cell viability in the EX group than in the PIO group. Several studies have also established that SIRT1 activation significantly attenuates cell apoptosis.^14^, ^18^ In agreement, our results indicated that PIO was protective on cisplatin‐treated samples and that this effect was achieved via SIRT1‐dependent suppression of cell apoptosis.

P53 is a substrate of SIRT1,^13^ which deacetylate its C‐terminal lysine residue after binding with SIRT1, leading to the inactivation of p53. This prevents the migration of p53 from the cytoplasm to the mitochondria, blocking the release of apoptotic proteins.^14^, ^15^, ^16^ Moreover, several studies have demonstrated that p53 plays an important role in the regulation of mitochondrial apoptosis and the cell cycle in cisplatin nephrotoxicity.^6^ The underlying mechanism involves deacetylation and acetylation of p53 by histone deacetylase (HDAC) and histone acetyl transferase, which regulates the permeability of mitochondrial outer membrane.^19^ In this study, we showed that PIO significantly suppressed the acetylation of p53 and promoted SIRT1‐p53 interaction after cisplatin treatment. These results indicated that anti‐apoptotic effect of SIRT1 activation is caused by the interaction between SIRT1 and p53, which inactivates p53 through deacetylation. These findings were in line with a previous study that showed that the inhibition of HDAC reduced cisplatin‐induced apoptosis of renal tubular cells by lessening cisplatin‐induced activation of p53.^14^

It has been shown that PUMA‐α is a modulator of apoptosis upregulated by p53.^7^ PUMA‐α interacts with Bcl‐2 family proteins, which are divided into three families: anti‐apoptotic proteins, apoptotic effector proteins and BH3‐only proteins.^16^ Bcl‐2 family proteins are the switches of mitochondrial apoptosis whose BH domains have a unique function.^16^ A special alpha helix in the structure of PUMA‐α interacts with the BH1, BH2 and BH3 domains on the surface of Bcl‐2 anti‐apoptotic proteins on the mitochondrial membrane to form hydrophobic grooves. This interaction relieves the inhibition of Bcl‐2 anti‐apoptotic protein (BIM, Bad) and activates Bax/Bak.^20^ PUMA‐α can also activate Bax/Bak by binding with apoptotic effector protein (Bax), which directly activates Bax, and by directly binding with anti‐apoptotic protein Bcl‐2 to direct pro‐apoptotic proteins.^17^, ^21^, ^22^ In this study, we showed that cisplatin significantly increased the Bax/Bcl‐2 ratio and upregulated the expression of Bax and PUMA‐α. Furthermore, cisplatin decreased Bcl‐2 expression in vivo and in vitro, demonstrating that cisplatin could induce the expression of PUMA‐α and apoptotic‐related proteins.^14^ SIRT1 activation by PIO suppressed this cisplatin‐induced change while inhibition of SIRT1 counteracted PIO, indicating that SIRT1 activation could suppress PUMA‐α‐mediated activation of Bax/Bak in cisplatin nephrotoxicity.

Studies have shown that activated Bax/Bak oligomerizes in the outer membrane of mitochondria, forming a channel pore or changing the original membrane channel protein. This leads to mitochondrial inner membrane permeabilization, ROS generation, mPTP opening, MMP dissipation, mitochondrial ATP synthesis cessation and ultimately cytochrome c release from mitochondria into the cytoplasm.^23^ Cytochrome c activates caspase‐9, leading to the downstream activation of executioner caspase‐3 to induce cell apoptosis.^17^ Our study demonstrated that PIO promoted mitochondrial ATP synthesis and suppressed the generation of ROS, opening of mPTP, dissipation of MMP, release of cytochrome c and activation of caspase‐3 and caspase‐9. These results indicated that SIRT1 activation could reduce cell apoptosis by suppressing mitochondrial abnormality in cisplatin nephrotoxicity. Notably, SIRT1 activation also regulates additional pathways other than the mitochondria/cytochrome c signalling to suppress the activation of the caspase cascade linking the endoplasmic reticulum pathway.^24^ Further studies to test the impact of SIRT1 activation on the endoplasmic reticulum pathway in mice are required.

In summary, our findings indicate that PIO prevents cisplatin nephrotoxicity by suppressing p53‐mediated mitochondrial apoptotic pathway via SIRT1 activation. This mechanism might provide clues for drug development targeting cisplatin nephrotoxicity. However, additional studies are necessary to further explore the mechanisms by which SIRT1 activation antagonizes cisplatin‐induced kidney damage.

## CONFLICT OF INTEREST

The authors declare there is no potential conflict of interest.

## AUTHOR CONTRIBUTION

**Jiong Zhang:** Writing‐original draft (equal). **Yang zou:** Data curation (equal). **Yan Cheng‐jing:** Supervision (equal). **Lu xiangheng:** Supervision (supporting). **Xue‐Peng Wang:** Supervision (equal). **Xiao‐Jia Yu:** Software (equal). **Guisen Li:** Conceptualization (equal). **JIa wang:** Conceptualization (equal).

## ETHICAL APPROVAL

The article is in compliance with ethical standards and authorized and approved by Sichuan province hospital ethics committee.

## Data Availability

The data that support the findings of this study are available from the corresponding author upon reasonable request.
